# Supplementary material for: Enhancing Professional Periodontal Therapy with a Novel PMA-Zeolite Application: A Clinical Study on Periodontal Outcomes and Microbiological Changes
Source: J Funct Biomater. 2025 Jul 22;16(8):270. doi: 10.3390/jfb16080270 (PMC12387170; doi:10.3390/jfb16080270)
Supplement: Supplementary file 1 [file jfb-16-00270-s001.zip › jfb-3686342-supplementary.pdf]

Supplementary material

Data on patients' drop-out

- 12 patients quitted the study in the very beginning during the recruiting, after the major earthquake in Zagreb in spring 2020 and did not come back for further examinations and follow-up even after direct telephone contacting.

- 13 patients did not comply with the treatment or quitted the treatment due to diagnosed Covid-19 disease or other declared 'private reasons' (only one patient specified the private reason as unexpected need for taking care of elderly family members).

- 1 patient did not provide clear data on treatment complying

- 5 patients did not show for control at the end of therapy (10th week)

- 6 control patients that received only standard periodontic prophylaxis, did not appear at the control examination after 10th week. Formally, these patients are noted as patients requiring further periodontic treatment. **The usual drop-out of such patients that do not return to control appointment, occurs due to lack of disease improvement (lack of treatment therapy effect) that increases fear of therapy continuation.**

Figure S1. Individual statistically relevant class differences in 7 patients. The two-sided Fisher's exact test with Benjamini-Hochberg FDR correction was used, p<0.05) showed a highly individualized response in class comparison due to large oral microbiome variations already documented in the literature. These individual data is presented for each patient.

Patient 1

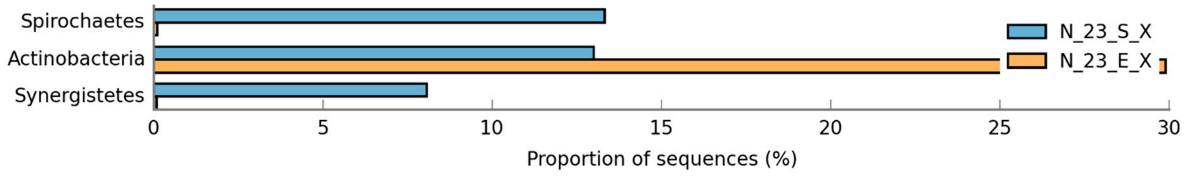

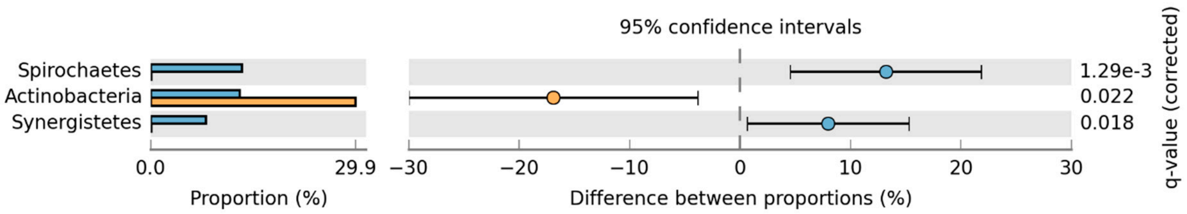

Patient 2

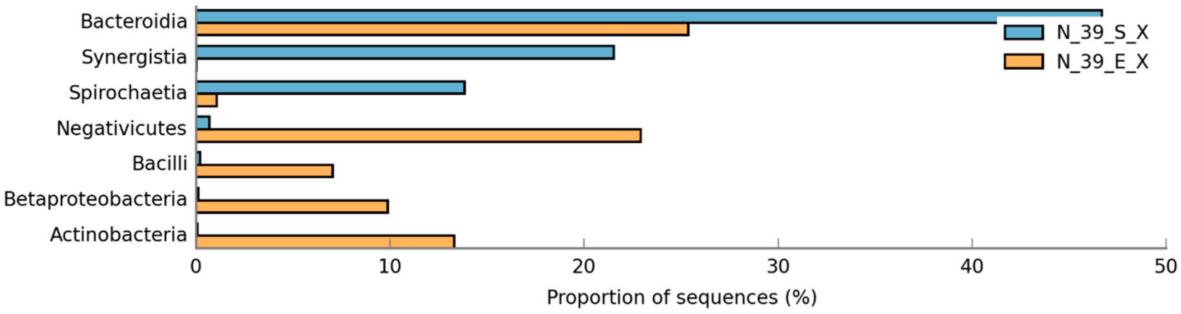

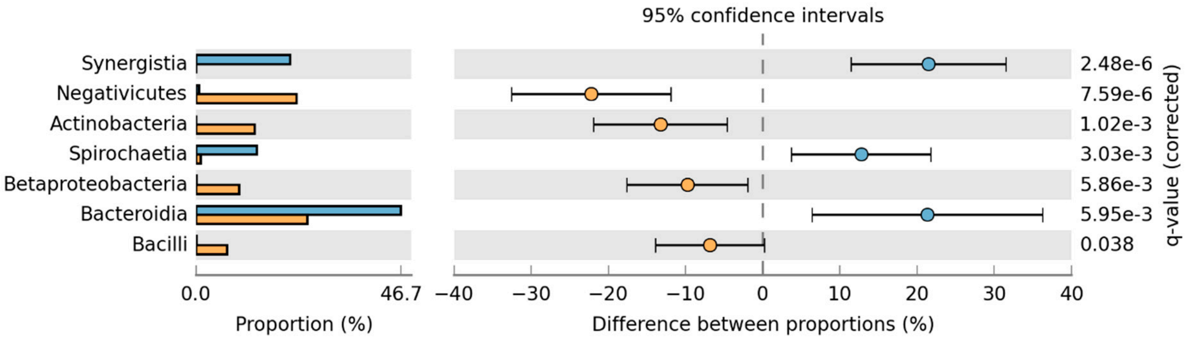

Patient 3

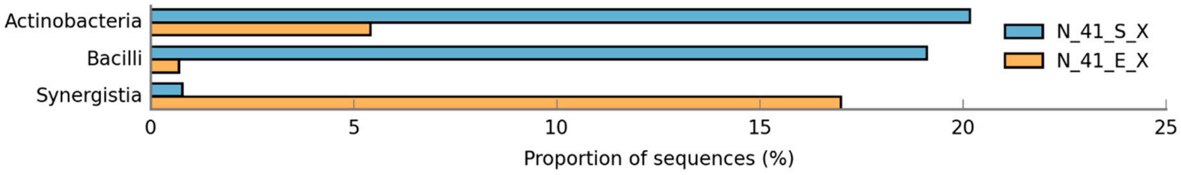

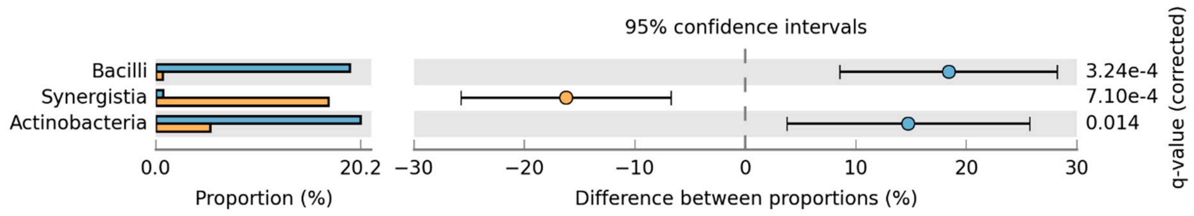
95% confidence intervals
Bacilli
Synergistia
Actinobacteria
3.24e-4
7.10e-4
0.014
q-value (corrected)
0.0
20.2
Proportion (%)
−30
−20
−10
0
10
20
30
Difference between proportions (%)

Patient 4:

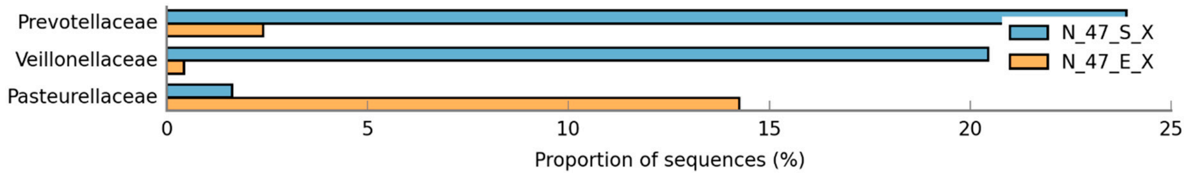
Prevotellaceae
Veillonellaceae
Pasteurellaceae
N_47_S_X
N_47_E_X
0
5
10
15
20
25
Proportion of sequences (%)

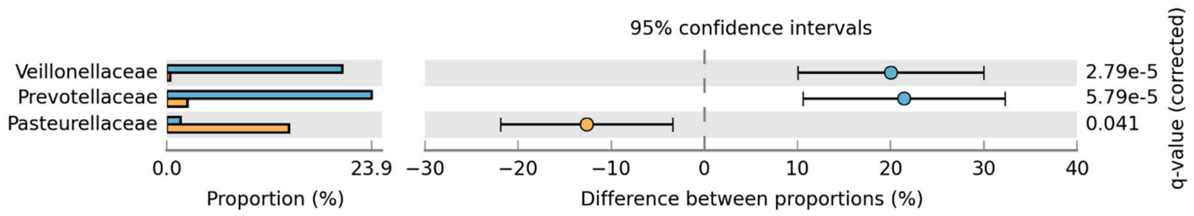
95% confidence intervals
Veillonellaceae
Prevotellaceae
Pasteurellaceae
2.79e-5
5.79e-5
0.041
q-value (corrected)
0.0
23.9
Proportion (%)
−30
−20
−10
0
10
20
30
40
Difference between proportions (%)

Patient 5:

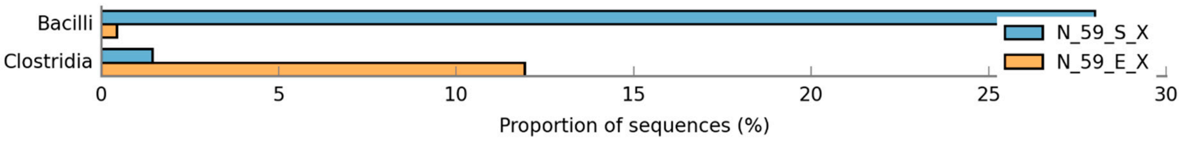

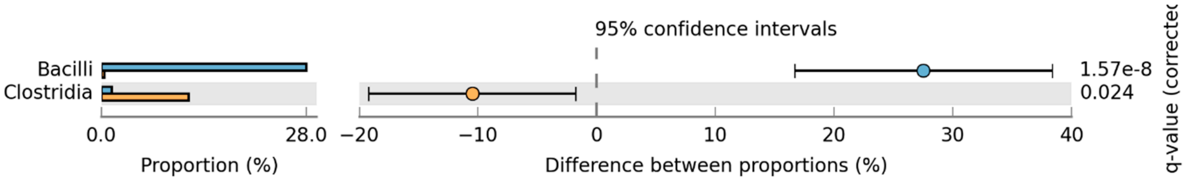

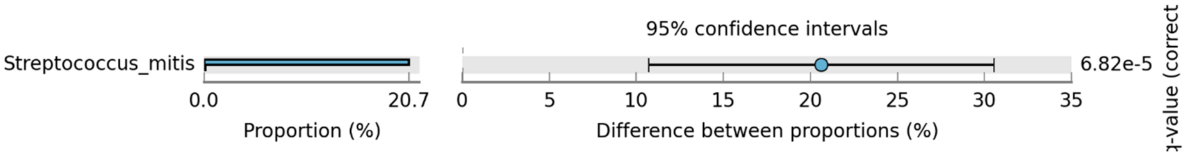

Patient 6

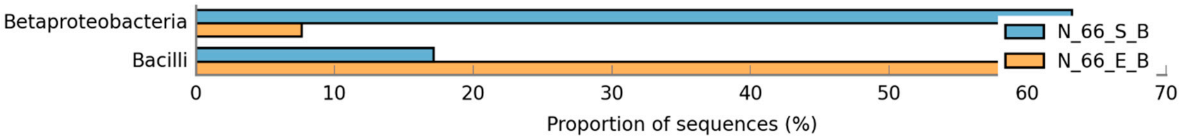

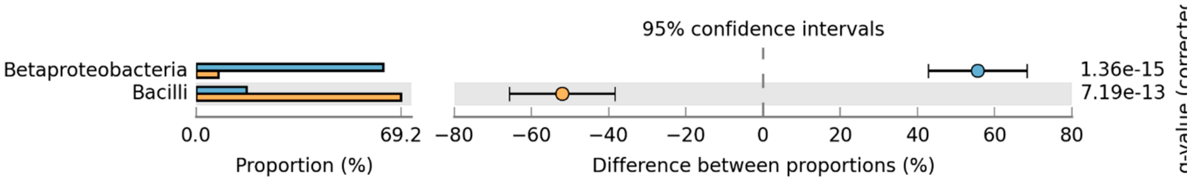

Patient 7

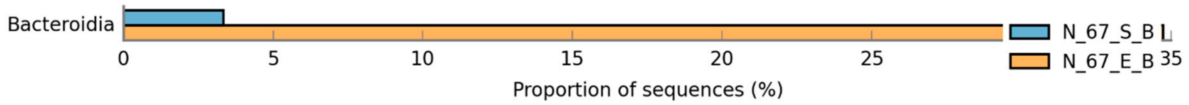
Bacteroidia
0
5
10
15
20
25
35
Proportion of sequences (%)
N_67_S_B
N_67_E_B

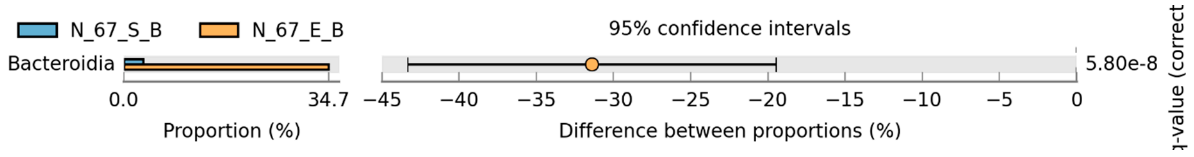
N_67_S_B
N_67_E_B
95% confidence intervals
Bacteroidia
5.80e-8
0.0
34.7
−45
−40
−35
−30
−25
−20
−15
−10
−5
0
Proportion (%)
Difference between proportions (%)
